# Development and validation of a model for predicting the risk of brain arteriovenous malformation rupture based on three-dimensional morphological features

**DOI:** 10.3389/fneur.2022.979014

**Published:** 2022-11-09

**Authors:** Shaosen Zhang, Shengjun Sun, Yuanren Zhai, Xiaochen Wang, Qian Zhang, Zhiyong Shi, Peicong Ge, Dong Zhang

**Affiliations:** ^1^Department of Neurosurgery, Beijing Tiantan Hospital, Capital Medical University, Beijing, China; ^2^Department of Radiology, Beijing Tiantan Hospital, Capital Medical University, Beijing, China

**Keywords:** brain arteriovenous malformation, three-dimensional morphological features, intracranial hemorrhage, nomogram, prediction model

## Abstract

**Objective:**

Brain arteriovenous malformation (bAVM) is an important reason for intracranial hemorrhage. This study aimed at developing and validating a model for predicting bAVMs rupture by using three-dimensional (3D) morphological features extracted from Computed Tomography (CT) angiography.

**Materials and methods:**

The prediction model was developed in a cohort consisting of 412 patients with bAVM between January 2010 and December 2020. All cases were partitioned into training and testing sets in the ratio of 7:3. Features were extracted from the 3D model built on CT angiography. Logistic regression was used to develop the model, with features selected using L1 Regularization, presented with a nomogram, and assessed with calibration curve, receiver operating characteristic (ROC) curve and decision curve analyze (DCA).

**Results:**

Significant variations in associated aneurysm, deep located, number of draining veins, type of venous drainage, deep drainage, drainage vein entrance diameter (Dv), type of feeding arteries, middle cerebral artery feeding, volume, Feret diameter, surface area, roundness, elongation, mean density (HU), and median density (HU) were found by univariate analysis (*p* < 0.05). The prediction model consisted of associated aneurysm, deep located, number of draining veins, deep drainage, Dv, volume, Feret diameter, surface area, mean density, and median density. The model showed good discrimination, with a C-index of 0.873 (95% CI, 0.791–0.931) in the training set and 0.754 (95% CI, 0.710–0.795) in the testing set.

**Conclusions:**

This study presented 3D morphological features could be conveniently used to predict hemorrhage from unruptured bAVMs.

## Introduction

Brain Arteriovenous malformation (bAVM), which is a congenital anomaly caused by capillary maldevelopment and shunts between brain arteries and veins, is an important cause of intracranial hemorrhage (1, 2). The risk of bAVM rupture is ~3% per year, and is considered as an important reason for intracranial hemorrhage in patients younger than 40 years (3–5). Many characteristics, such as size, deep located, venous drainage pattern, fewer draining veins, and feeding arteries, have been described as risk factors of bAVM rupture (3, 5–12). The hemorrhage due to bAVM causes serious consequences and has a higher risk of hematoma removal through surgery due to the nidus (2), making it important to predict hemorrhage before rupture.

Several authors reported that small bAVMs had a higher risk of rupture (13–15), but another study showed that large bAVMs bled more frequently (12). BAVMs have irregular geometries, but the size of bAVMs have been commonly measured by the diameter of the maximum cross-section on CT or Magnetic Resonance (MR) images, which could not describe the nidus exactly. The morphological features extracted by the 3D model can more accurately describe the bAVMs. Otherwise, there are various risk factors with angio-architectural features to predict bAVM rupture; however, the weight of these features may become different when combined with the 3D morphological features.

In this study, we evaluated different angio-architectural features of bAVM on CT angiography in a single-institute cohort to investigate the key points. First, we used 3D model instead of the tomographic images to evaluate the effect of size in predicting hemorrhage. Second, we evaluated the effect of other 3D morphological features and built a nomogram consisting of architectural features for clinical usage.

## Materials and methods

### Patients and image materials

All patients were diagnosed with bAVM between January 2010 and December 2020 in multiple centers, including Taiyuan Central Hospital of Shanxi Medical University, Zhumadian Central Hospital, The First People's Hospital of Lianyungang, Shanghai Changhang Hospital, and Ordos Central Hospital, and were retrospectively included in this study.

The following were the inclusion and exclusion criteria. Inclusion criteria: diagnosis of unruptured bAVM using Digital subtraction angiography (DSA) in brain, basal ganglia, thalamus, corpus callosum, cerebellum and other locations, intracranial hemorrhage associated with bAVM diagnosed using CT imaging, and CT angiography images acquired before rupture and without surgical or interventional treatment prior to acquisition. Exclusion criteria: simple arteriovenous fistula, combined Dural arteriovenous fistula, and occurrence of lesions in the spinal cord. Only patients with CT angiography data on initial hemorrhage status were included.

All CT data acquisition through the same type of equipment (Revolution EVO; GE Healthcare). The scan protocols were as follows: axial plane; 80 kVp; automatic tube current modulation, 300–580 mA; section thickness, 0.625 mm; collimation, 80 mm; rotation time, 0.5 s; noise index, 2.2.

This study was approved by the ethics committee of Beijing Tiantan Hospital (ID: KY-2017-068-02). All patients provided signed informed consent.

### 3D model building and definition of 3D morphological features

**Figure 1:**
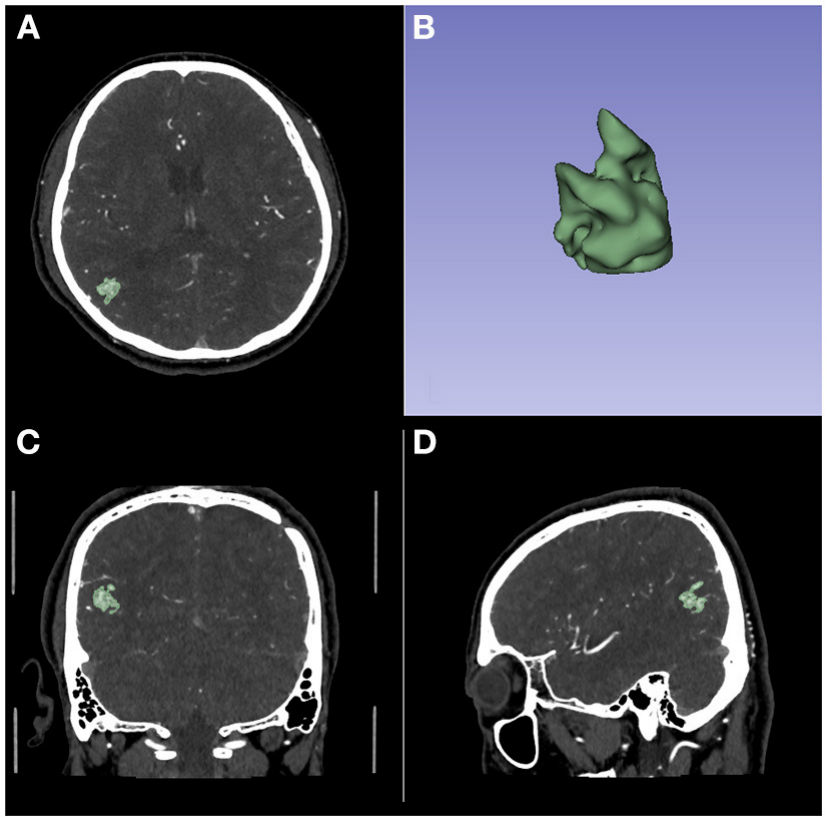
An example of 3D model building on CT Angiography image. **(A)** The axial image. **(B)** The 3D model. **(C)** The coronal image. **(D)** The sagittal image.

The 3D models were built on CT angiography using the 3D Slicer software (version 4.11, https://www.slicer.org). Each case was segmented in axial view and corrected in sagittal and coronal views by three researchers. All the three researchers had at least 5 years related working experience. The quality of the model was checked by other two researchers. Researchers participating in the review had at least 10 years of related working experience. See Figure 1 for an example of 3D model building.

The features collected from the models included Feret diameters (cm), volume (cm^3^), surface area (cm^2^), roundness, flatness, elongation, mean density (HU), median density (HU), and standard deviation of density (HU). None of the features included the portion of the draining vein away from the nidus.

All the nidus was completely segmented and the features were all based on the entire nidus. Feret diameter is the diameter of a sphere that encompasses the entire segment. The surface area is the volume of the segment, including the outside surface and the surface of the interior hollowed-out parts. Roundness was calculated from the ratio of the area of the sphere calculated from the Feret diameter by the actual area. Flatness is calculated from the square root of the ratio of the second smallest principal moment to the smallest. Elongation is calculated from the square root of the ratio of the second largest principal moment to the second smallest. All features were calculated using the 3D Slicer software.

### Clinical features selection

With the exception of the features collected from the 3D model, other features were selected based on previous literature reports and clinical experiences (3, 6, 7). The features included associated aneurysm, calcification, location, features of venous drainage, and features of feeding arteries.

The associated aneurysm included feeding artery aneurysm and intranidal aneurysm. Arterial aneurysms were defined as saccular dilatations of the lumen more than 2 times the width of the arterial vessel that carried the dilatation which located in the feeding arteries or inside the nidus (9). Arterial aneurysms located on the arteries not contributing blood flow to the bAVM were considered unrelated to the bAVM and were not included in the analysis.

Locations were grouped into frontal, temporal, parietal, occipital, and deep located. The deep located nidus was defined as the nidus that occurred in the basal ganglia, thalamus, and corpus callosum, and was recorded as 0 in the number of affected lobes. The location indicated that all the nidus or a part of it was located in some lobes.

The features of venous drainage included the number of draining veins, the different combination types of venous drainage and the drainage vein entrance diameter. The venous drainage was grouped into the superior sagittal sinus, transverse sinus and sigmoid sinus, and straight sinus. This was only recorded if the case had the draining vein to each sinus, which meant that a case could drain to several sinuses. The combination types were classified as extensive superficial sinus combining straight sinus drainage, extensive superficial sinus, drainage, superior sagittal sinus combined with straight sinus drainage, transverse sinus and sigmoid sinus combined with straight sinus drainage, straight sinus drainage, and single superficial sinus drainage. The drainage vein entrance diameter (Dv) was also recorded. The entrance stenosis of the draining vein had been shown to be a risk factor for bAVM rupture in previous studies (9, 13, 16), and the diameter of the entrance of the draining vein was a quantitative assessment of the entrance stenosis of the draining vein.

The feeding arteries were grouped into the anterior cerebral artery (ACA), middle cerebral artery (MCA), and posterior cerebral artery (PCA). Different combinations of feeding arteries were also recorded. The combination of feeding arteries was grouped into ACA combined with MCA and PCA, ACA combined with MCA, ACA combined with PCA, MCA combined with PCA, and single artery feeding.

### Statistical analysis

Statistical analyses were conducted using R software (version 4.1.0; http://www.Rproject.org). In univariate analysis, the Pearson chi-square test was used to compare the counting data. If measurement data were normally distributed, the *t*-test was used for comparison; otherwise, the rank sum test was used. Features with significant differences (*p* < 0.05) in univariate analysis between the two groups were included in the multivariate regression. The Kolmogorov–Smirnov (K–S) test was utilized to examine the normality of continuous features. Age, volume, Feret diameter, surface area, roundness, elongation, mean density, median density, and standard deviation of density were all skewed in this study. Based on the distribution of the data, the Mann-Whitney *U* test was chosen as the approach to compare the two groups.

Prior to model training, all non-normally distributed continuous variables were transformed into normally distributed variables by power transformation. All normally distributed variables were normalized by the zero-mean method, and all categorical variables were transformed into one-hot codes.

All cases were partitioned into training and testing sets in the ratio of 7:3. The model was built using logistic regression, and the model downscaling and feature filtering methods were L1 regularization. Logistic regression is a multi-factor statistical method of analysis that is highly interpretable. Logistic regression analysis results can be displayed using a nomogram, making clinical use easier (17–19). The reported statistical significance levels were all two-sided, with the significance set at 0.05.

### Model demonstration and validation

Using the results of logistic regression, we developed a mathematical model with strong interpretability and demonstrated it by nomogram for predicting bAVM rupture, which was a quantitative tool for clinical use.

Validation of the nomogram included internal and external validations. The internal validation used calibration curve. External validation used ROC curve in both the training and validation set. The performance of clinical use of the nomogram was quantified by decision curve analysis in both the training and testing. As a reference, we compared the predictive ability of Spetzler-Martin score for hemorrhage in this part.

## Results

### Baseline information

In total, 581 patients were identified and comprised the cohort: 332 males and 249 females; mean age of 34.09 ± 16.04 years, ranging from 4 to 79 years; 206 hemorrhage and 375 non-hemorrhage cases.

The effect of age and sex on the prediction of bAVM rupture was not clear (16, 20). To focus on the effect of radiologic features, propensity score matching (PSM) was carried out based on the baseline of the hemorrhage group, with a ratio of 1:1. The training cohort finally contained 412 patients, with 206 males and 206 females, 206 hemorrhage and 206 non-hemorrhage cases, and a mean age of 31.72 ± 16.35 years, ranging from 4 to 79 years.

The cohort included 206 hemorrhage cases, and 206 non-hemorrhage cases were matched and extracted into the cohort. The hemorrhage group included 114 male (55.3%) and 92 female patients (44.7%), while the non-hemorrhage group included 113 male (54.9%) and 93 female patients (45.1%). The median age of the hemorrhage group was 30 (21–45) years, and that of the non-hemorrhage group was 27 (18–42) years. There was no significant difference between the non-hemorrhage and hemorrhage groups (see Table 1). Since Spetzler-Martin score was calculated by several basic characteristics, it was not included in subsequent statistics as an independent risk factor.

**Table 1:** The baseline information after preference score matching.

	**Non-hemorrhage (*n* = 206)**	**Hemorrhage (*n* = 206)**	***p*-value**
Sex			0.921
Male	114 (55.3%)	92 (44.7%)	
Female	113 (54.9%)	93 (45.1%)	
Age	31 (21–45)	27 (18–42)	0.169
Spetzler-martin			0.563
Score			
2	23 (11.2%)	30 (14.6%)	
3	73 (35.4%)	88 (42.7%)	
4	78 (37.9%)	66 (32.0%)	
5	32 (15.5%)	22 (10.7%)	

### Results of univariate analysis

All the characteristics collected from the CT images and 3D models are presented in Table 2. Volume, Feret diameter, surface area, roundness, elongation, flatness, mean density, and median density were under a skewed distribution and showed with quartiles in Table 2. Significant variations were observed in associated aneurysm, deep located, number of draining veins, type of venous drainage, deep drainage, Dv, type of feeding arteries, MCA feeding, volume, Feret diameter, surface area, roundness, elongation, mean density, and median density (*p* < 0.05).

**Table 2:** The results of univariate analysis.

	**Non-hemorrhage (*n* = 206)**	**Hemorrhage (*n* = 206)**	***p*-value**
Associated aneurysm	38 (18.5%)	59 (28.7%)	< 0.001
Feeding artery aneurysm	14 (6.8%)	22 (10.7%)	
Intranidal aneurysm	24 (11.7%)	37 (18.0%)	
Calcification	48 (23.3%)	37 (18.0%)	0.180
Number of affected lobes			0.062
0	14 (6.8%)	31 (15.0%)	
1	129 (62.6%)	115 (55.8%)	
2	54 (26.2%)	52 (25.2%)	
3	9 (4.4%)	8 (3.9%)	
Frontal	90 (43.7%)	79 (38.3%)	0.271
Parietal	64 (31.1%)	69 (33.5%)	0.598
Temporal	72 (35.0%)	64 (31.1%)	0.402
Occipital	38 (18.4%)	31 (15.0%)	0.356
Deep located	14 (6.8%)	31 (15.0%)	0.007
Number of draining veins			0.003
1	146 (70.9%)	166 (80.6%)	
2	33 (16.0%)	32 (15.5%)	
≥3	27 (13.1%)	8 (3.9%)	
Type of venous drainage			< 0.001
Extensive superficial drainage + straight sinus	11 (5.3%)	0	
Extensive superficial drainage	12 (5.8%)	16 (7.8%)	
Superior sagittal sinus + straight sinus	17 (8.3%)	16 (7.8%)	
Transverse sinus + straight sinus	6 (2.9%)	5 (2.4%)	
Straight sinus	26 (12.6%)	53 (25.7%)	
Single superficial sinus	134 (65.0%)	116 (56.3%)	
Superior sagittal sinus drainage	142 (68.9%)	126 (61.2%)	0.098
Transverse sinus drainage	61 (29.6%)	43 (20.9%)	0.141
Deep drainage	60 (29.1%)	74 (35.9%)	0.041
Dv (cm)	0.65 (0.50–0.84)	0.49 (0.39–0.67)	< 0.001
Type of feeding arteries			0.008
ACA + MCA + PCA	12 (5.8%)	3 (1.5%)	
ACA + MCA	33 (16.0%)	17 (8.3%)	
ACA + PCA	4 (1.9%)	5 (2.4%)	
MCA + PCA	25 (12.1%)	21 (10.2%)	
Single artery feeding	132 (64.1%)	160 (77.7%)	
ACA feeding	71 (34.5%)	65 (31.6%)	0.530
MCA feeding	149 (72.3%)	120 (58.3%)	0.003
PCA feeding	72 (35.0%)	70 (34.0%)	0.836
Volume (cm^3^)	9.95 (3.44–24.27)	3.89 (1.67–12.62)	< 0.001
Feret diameter (cm)	5.35 (3.88–7.69)	4.49 (3.33–6.03)	< 0.001
Surface area (cm^2^)	78.53 (32.24–168.87)	39.79 (16.05–90.38)	< 0.001
Roundness	0.29 (0.24–0.39)	0.34 (0.28–0.43)	< 0.001
Flatness	1.31 (1.21–1.52)	1.37 (1.21–1.54)	0.177
Elongation	1.38 (1.21–1.64)	1.44 (1.27–1.78)	0.009
Mean density (HU)	306.91 (251.48–350.04)	269.57 (232.00–327.74)	0.001
Median density (HU)	288.50 (236.00–338.25)	243.50 (193.75–311.00)	< 0.001
Standard deviation of density (HU)	129.40 (91.74–159.93)	124.18 (101.69–154.95)	0.956

### Feature selection and development of the prediction model

All the characteristics which showed significant variation in the univariate analysis were extracted into the logistic regression model. Associated aneurysm, volume, surface area, mean density, and median density were identified as independent predictors under L1 Regularization. Deep located, number of draining veins, deep drainage, Dv, Feret diameters, which had been classically described in high quality prospective AVM natural history studies as high-risk factors for hemorrhage (2, 7, 9), were also contained in the final model (see Table 3). Logistic regression revealed that associated aneurysm, deep located, number of draining veins, and deep drainage were risk factors for bAVM rupture. These risk factors increased the probability of a ruptured bAVM in direct proportion to their existence. Intranidal aneurysm exhibited a higher probability of rupture than feeding artery aneurysm among associated aneurysms. The risk of bAVM rupture rose with decreasing Feret diameter, increasing volume, decreasing surface area, increasing mean density, and decreasing median density, as evidenced by the results for characteristics whose attributes were continuous variables.

**Table 3:** The risk factors of hemorrhage caused by AVM rupture extracted into logistic regression after filtration.

	**OR**	**95%CI**	***p*-value**
Associated aneurysm	3.276	2.061–5.206	0.000
Deep located	2.036	0.907–4.567	0.085
Number of draining veins	1.045	0.642–1.702	0.858
Deep drainage	1.120	0.653–1.923	0.680
Dv (cm)	0.355	0.128–0.989	0.048
Volume (cm^3^)	1.089	1.033–1.148	0.002
Feret diameters (cm)	0.948	0.780–1.152	0.591
Surface area (cm^2^)	0.983	0.973–0.992	0.000
Mean density (HU)	1.014	1.001–1.027	0.003
Median density (HU)	0.983	0.971–0.995	0.005

While the weights of other variables were lower, the relative weights of the associated aneurysm, Dv, volume, surface area, mean density, and median density were higher. Other characteristics had varying weights as well. Because of normalization, these feature weights had a specific comparative value. The weights represented the importance of these features for this dataset and, as a result, the intensity of their influence on the bAVM rupture. As a proper set of additional factors to the traditional bAVM rupture risk factors, 3D features were weighted more highly in the model.

**Figure 2:**
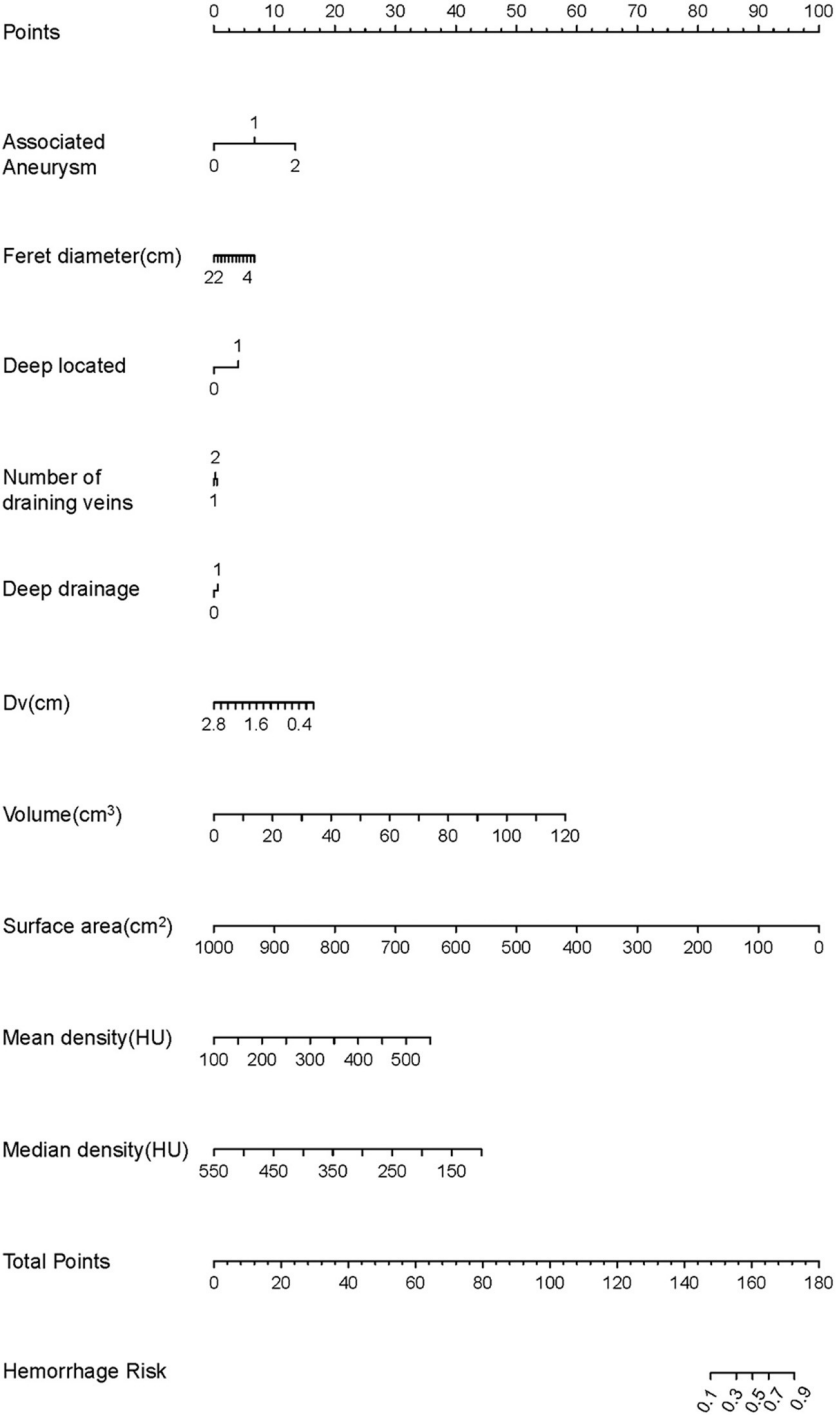
The development of the nomogram. The features are ranked by the standard deviation of the βx's. Every factor can match a score using the nomogram. The total score can correspond to the predicted value of the hemorrhage risk on a scale.

A nomogram was employed to illustrate the model in order to boost its therapeutic utility (Figure 2). The nomogram allowed each risk factor to be assigned a corresponding point score, and the overall score was used to calculate the associated probability of rupture.

### Validation of the prediction model

**Figure 3:**
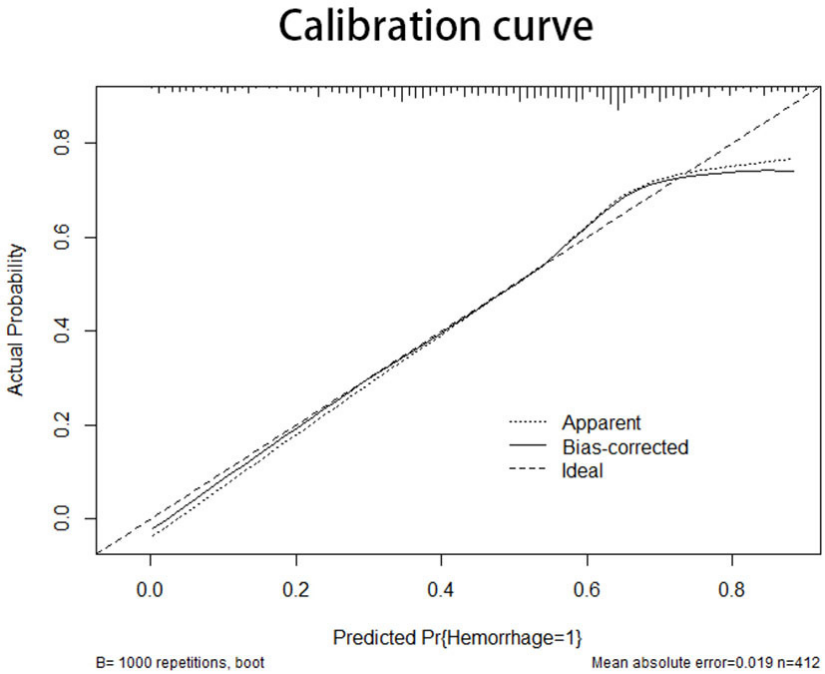
The X-axis of the calibration curve shows the predicted probability, and the Y-axis shows the actual probability. The calibration curve represents the performance of the nomogram in internal validation. The diagonal line represents the ideal model of prediction; the closer the curve is fitted to the diagonal line, the better the model performs.

The information for the training set and test set was shown in Table 4. The calibration curve demonstrated good agreement between the predicted probability and actual probability; the figure of the calibration curve is shown in the supplementary file. The result of the Hosmer–Lemeshow test showed a significance of *p* > 0.05 (Figure 3).

**Table 4:** The features of the training set and testing set.

	**Training set**		**Testing set**	
	**Non-hemorrhage (*n* = 144)**	**Hemorrhage (*n* = 144)**	**Non-hemorrhage (*n* = 62)**	**Hemorrhage (*n* = 62)**
**Sex**
Male	72 (50.0%)	87 (60.4%)	42 (67.7%)	26 (41.9%)
Female	72 (50.0%)	57 (39.6%)	20 (32.3%)	36 (58.1%)
Age	29 (19–45)	27 (18–43)	33 (22–46)	26 (18–40)
**Spetzler-martin score**
2	18 (12.5%)	18 (12.5%)	5 (8.1%)	12 (19.4%)
3	51 (35.4%)	64 (44.4%)	22 (35.5%)	24 (38.7%)
4	51 (35.4%)	48 (33.3%)	27 (43.5%)	18 (29.0%)
5	24 (16.7%)	14 (9.7%)	8 (12.9%)	8 (12.9%)
Associated aneurysm	30 (20.8%)	40 (27.8%)	8 (12.9%)	19 (30.6%)
Feeding artery aneurysm	18 (12.5%)	26 (18.1%)	6 (9.7%)	11 (17.7%)
Intranidal aneurysm	12 (8.3%)	14 (9.7%)	2 (3.2%)	8 (12.9%)
Deep located	10 (6.9%)	18 (12.5%)	4 (6.5%)	13 (21.0%)
**Number of draining veins**
1	100 (69.4%)	115 (79.9%)	46 (74.2%)	51 (82.3%)
2	23 (16.0%)	24 (16.7%)	10 (16.1%)	8 (12.9%)
≥3	21 (14.6%)	5 (3.5%)	6 (9.7%)	3 (4.8%)
Deep drainage	44 (30.6%)	45 (31.3%)	16 (25.8%)	29 (46.8%)
Dv (cm)	0.61 (0.50–0.82)	0.50 (0.39–0.68)	0.68 (0.50–0.90)	0.47 (0.38–0.64)
Feret diameter (cm)	5.30 (3.67–7.43)	4.52 (3.41–5.99)	4.41 (4.11–8.00)	4.29 (2.90–6.15)
Volume (cm^3^)	9.46 (3.18–23.75)	4.14 (1.73–13.07)	10.86 (3.93–27.37)	3.68 (1.49–12.61)
Surface area (cm^2^)	74.77 (31.09–163.81)	39.79 (17.08–94.84)	84.92 (33.11–186.73)	38.95 (12.76–81.23)
Mean density (HU)	308.31 (256.16–353.36)	267.43 (225.65–321.03)	294.93 (237.87–349.14)	275.18 (244.95–331.98)
Median density (HU)	293.50 (236.75–348.50)	243.50 (192.00–301.75)	278.50 (235.00–322.25)	243.50 (200.00–314.00)

The characteristics include baseline information and all the features of the prediction model.

**Figure 4:**
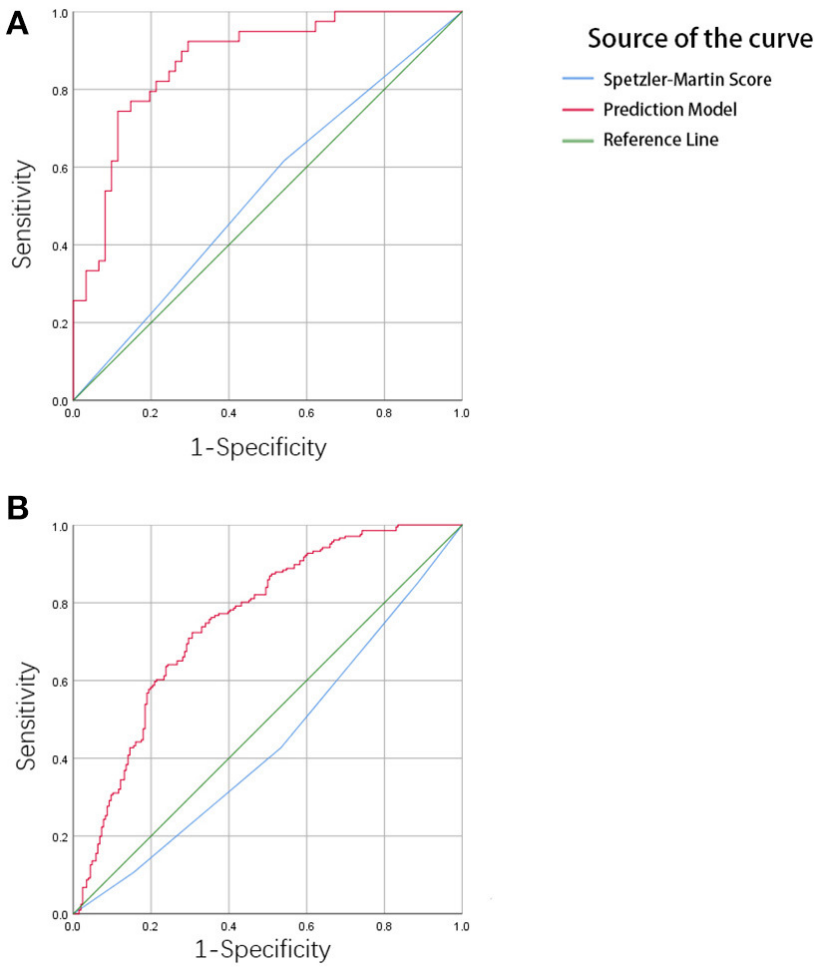
**(A)** The result of ROC analysis in the training cohort. The area under the curve (AUC) is 0.873, *p* < 0.001. **(B)** The result of ROC analysis in the validation cohort. The area under the curve is 0.754, *p* < 0.001.

In the training set, the C-index of the prediction model was 0.873 with a 95% CI of 0.791–0.931 (*p* < 0.001), sensitivity was 76.92, and specificity was 90.16. In the testing set, the C-index of the prediction model was 0.754 with a 95% confidence interval (CI) of 0.710–0.795 (*p* < 0.001), sensitivity was 72.33, specificity was 69.42. The Speztler-Martin score's ability to predict hemorrhage was also evaluated and compared with the prediction model. The C-index of Speztler-Martin Score was 0.563 in training set and 0.440 in testing set. The ROC curve was shown in Figure 4.

### Clinical use

**Figure 5:**
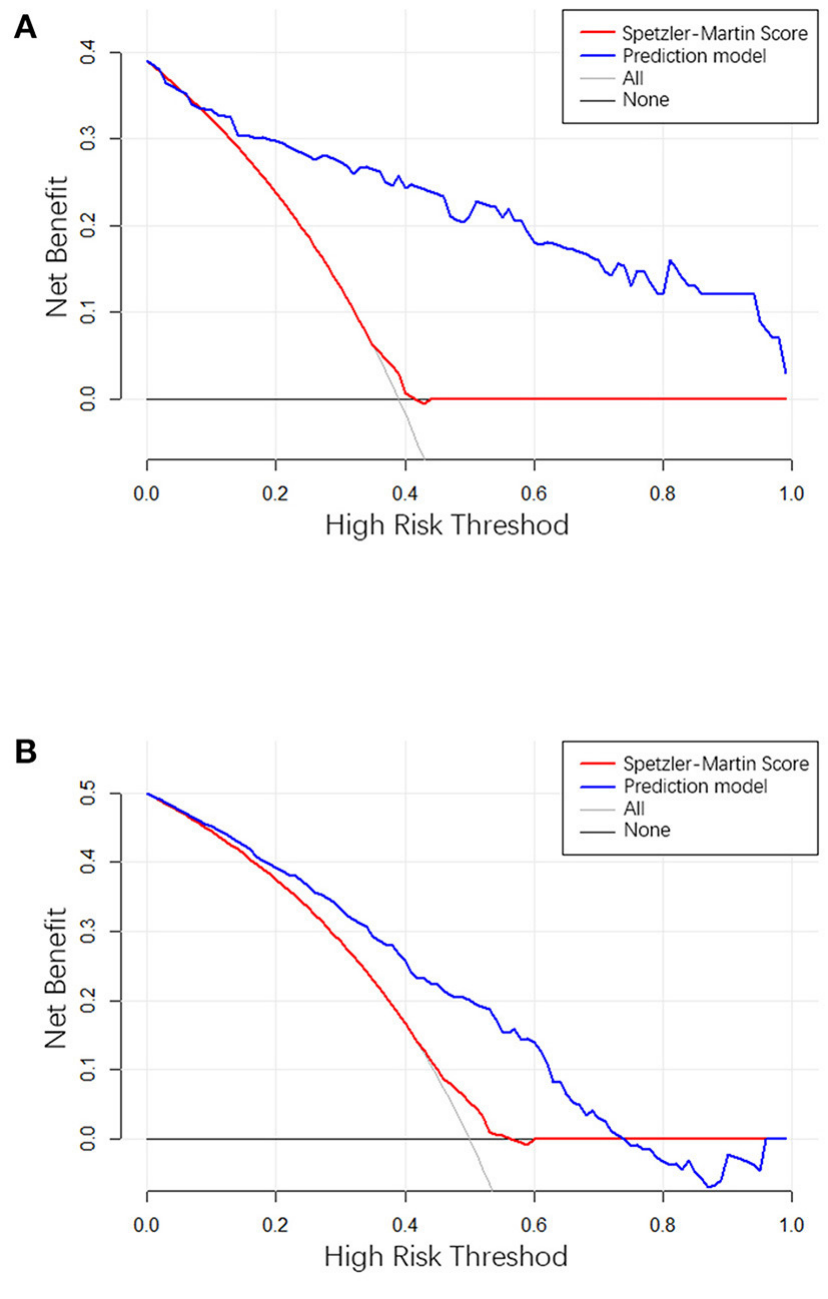
**(A)** The result of decision curve analysis in the training cohort. **(B)** The result of decision curve analysis in the validation cohort. The X-axis is the high-hemorrhage risk threshold, and Y-axis is the net benefit. The red line represents the prediction model. The net benefit is calculated by subtracting the proportion of all patients who are false positive from the proportion who are true positive, weighted by the relative harm of forgoing treatment compared with the negative consequences of an unnecessary treatment. The oblique line in the figure represents the net benefit when all the patients are treated as having hemorrhage, and gray horizontal line represents the net benefit when all the patients are treated as belonging to the non-hemorrhage group.

The clinical use of the prediction model was evaluated using the decision curve, as shown in Figure 5. The decision curve showed the net benefit of the prediction model, which was much better than the all-treated or none-treated approach, and was usable not only in the training set, but also in the testing set. The Speztler-Martin score was also evaluated using the decision curve and compared with the prediction model. The result showed that the prediction model performed well and was useful in clinic.

The actual effectiveness of the model in clinical practice was shown by both the ROC curve and the DCA curve; the higher the performance, the higher the usability. However, the performance of the testing set was more important for the model because these results demonstrate the model's dependability.

## Discussion

This study developed and validated a nomogram for predicting hemorrhage caused by unruptured AVMs. The nomogram makes the prediction model easy to use clinically. Doctors can use the 3D Slicer or other model-building software to segment the bAVM and export the features for prediction, which can then be used to calculate the probability of hemorrhage. The results showed that the prediction model performed well in both training set and testing set. In bAVM patients with medical treatment alone, prediction of the risk of hemorrhage is important, and the model of hemorrhage risk evaluation by 3D morphological parameters is an important addition to the traditional risk factors, while all risk factors are easy to understand and observe. In choosing the patient group for this study, no specific homogeneous population was considered. However, this study was balanced by PSM between groups of included patients to verify that there was no unnecessary bias in population age and sex.

The risk factors for hemorrhage from bAVMs have been a matter of discussion in the literature. In the early years, the viewpoints of authors were that the smaller bAVMs ruptured more frequently (14, 17, 18). As more studies were carried out, the conclusion for the relation between size and hemorrhage due to bAVMs changed. Macro et al. mentioned in their study that large bAVMs were more likely to cause hemorrhage, and other studies concluded that size was not a risk factor (10, 19, 21, 22). In clinical experience, different sizes often lead to different risks of hemorrhage, and the argument should come from the method of size measurement. The size of bAVMs was commonly measured in the CT or DSA images in one position, which could not accurately evaluate the nidus. Different researchers might measure the same nidus differently, which might lead to bias in the study results. In this study, the size of bAVMs was measured in several features using a 3D model, including the Feret diameter, volume, and the number of affected lobes. It was difficult to describe the bAVM as large or small because the variety of bAVM shapes are only defined by using the diameter of the maximum cross-section. From the results of the study, it was observed that although the Feret diameter was more accurate compared to the conventional diameter measurement, the weight of Feret diameter was still not as high as the volume, indicating that volume was a more informative indicator in the description of nidus size.

Research in recent years has shown that associated aneurysms including feeding artery aneurysms and intranidal aneurysms significantly increased the risk of hemorrhage (23, 24), but the effect of this factor was hard to evaluated. Through the nomogram, the weight of associated aneurysm in predicting hemorrhage can be quantitative. The individual score of feeding artery aneurysm and intranidal aneurysm are all more than 70. The score of feeding artery aneurysm is higher than intranidal aneurysm, which means higher risk of hemorrhage.

The surface area is another important factor relative to hemorrhage. No study has mentioned that the surface area is a risk factor. This is because the surface area can only be measured using a 3D model. The surface area has not been a feature to evaluate the size of bAVMs. The nidus of bAVM is a cellular structure (25), and the surface area is a feature that describes the CT value degree of the nidus. The smaller the surface area at the same volume, the closer the lesion is to a sphere, implying a more compact lesion structure, which quantifies to some extent the traditional description of lesion dispersion. At the same time, when the lesion structure is sparser, there are more hollow parts within the nidus, which also increases the surface area of the lesion. This study found that a smaller surface area was a risk factor for hemorrhage, which means that a closer nidus structure is more likely to rupture.

The CT density value of the nidus is also a risk factor. The density value represents different components of the nidus. Similar to the surface area and volume, the CT density of the nidus can only be measured completely using the 3D model. The results showed that the mean density and median density had different effects in predicting hemorrhage. The results of this study show that a higher mean density and lower median density lead to higher risk. This means that the nidus with a CT density under a positively skewed distribution has a higher risk of hemorrhage. The positively skewed distribution has another characteristic that the mode is focused on the left side of the mean, which means that this type of bAVMs have more venous components.

The method of drainage is also an important part of the discussion in the literature. The number of draining veins, deep drainage and draining vein stenosis have been recognized as risk factors for hemorrhage (11, 21, 26). In this study, a single drainage vein, deep drainage, and smaller drainage vein entrance diameter were all independent risk factors for bAVM rupture, which is consistent with the findings of currently available studies. Meanwhile, for the present data set, the most important predictor of hemorrhage risk was the entrance diameter of the drainage vein, a feature that was equivalent to the quantification of drainage vein stenosis. When the draining vein is thin, the presence of an entrance stenosis is difficult to observe, and measuring the entrance diameter may be a fairly simple method that proves to be equally effective.

The prediction model of this study was validated by both internal and external validations. The calibration curve represents the results of the internal validation (27). The curve had a fine degree of fitting compared with the ideal curve, but had a tendency to overestimate the risk of hemorrhage when the predicted probability was more than 0.7.

The ROC curve shows the results of the external validation. The C-index of the model was 0.873 and 0.754 in the training and testing set, respectively. This result showed that the discrimination of the nomogram was satisfactory. The decision curve can justify the clinical usefulness of the model through the net benefit compared to the all-treated and none-treated groups (28). The results of the decision curve analysis showed that the net benefit of the prediction model was better than both the all-treated and none-treated in the training and testing set. Due to the natural history and ethical requirements of bAVM itself, a multi-institutional prospective validation of the nomogram was not suitable.

This study had some limitations. The database of this study did not include bAVM located in brainstem, but the location was not a factor for predicting hemorrhage according to the results. This work was a retrospective study, and some unpredictable bias in case inclusion was inevitable. In this study, all cases met the same inclusion criteria and the equipment and parameters for image acquisition were strictly standardized. All image data were quality verified by experienced radiologists. Features such as deep located, deep drainage, Number of draining veins, although included in the final model, appeared to have limited feature weights, which may be due to the data distribution of the data set applied in this experiment and did not indicate that these features were not important in the prediction of bAVM rupture. Also, the degree of dispersion which considered in previous studies as potentially influencing the prognosis of bAVM were not included as a feature because they could not be quantified, but we believe that the role of 3D morphological features in predicting bAVM hemorrhage can be better assessed by more objective metrics such as volume and surface area.

## Data availability statement

The raw data supporting the conclusions of this article will be made available by the authors, without undue reservation.

## Ethics statement

The studies involving human participants were reviewed and approved by Ethics Committee of Beijing Tiantan Hospital. Written informed consent to participate in this study was provided by the participants' legal guardian/next of kin.

## Author contributions

DZ and SZ conceived and designed the study. SZ, YZ, and XW segmented the nidus and organized the case information. SS and QZ confirmed the quality of the cases and the segmentation. SZ, QZ, ZS, and PG performed statistical analyses. DZ, SZ, and SS contributed to the interpretation of the results. SZ drafted the manuscript for publication. All authors have critically revised the manuscript.

## Conflict of interest

The authors declare that the research was conducted in the absence of any commercial or financial relationships that could be construed as a potential conflict of interest.

## Publisher's note

All claims expressed in this article are solely those of the authors and do not necessarily represent those of their affiliated organizations, or those of the publisher, the editors and the reviewers. Any product that may be evaluated in this article, or claim that may be made by its manufacturer, is not guaranteed or endorsed by the publisher.
