# Corrigendum: Development and validation of a model for predicting the risk of brain arteriovenous malformation rupture based on three-dimensional morphological features

**DOI:** 10.3389/fneur.2023.1227701

**Published:** 2023-06-06

**Authors:** Shaosen Zhang, Shengjun Sun, Yuanren Zhai, Xiaochen Wang, Qian Zhang, Zhiyong Shi, Peicong Ge, Dong Zhang

**Affiliations:** ^1^Department of Neurosurgery, Beijing Tiantan Hospital, Capital Medical University, Beijing, China; ^2^Department of Radiology, Beijing Tiantan Hospital, Capital Medical University, Beijing, China

**Keywords:** brain arteriovenous malformation, three-dimensional morphological features, intracranial hemorrhage, nomogram, prediction model

In the published article, there was an error in affiliations 1, 2. Instead of “Beijing Tiantan Hospital, Beijing, China”, it should be “Beijing Tiantan Hospital, Capital Medical University, Beijing, China”.

The authors apologize for this error and state that this does not change the scientific conclusions of the article in any way. The original article has been updated.

